# Visitation of artificial watering points by the red fox (*Vulpes vulpes*) in semiarid Australia

**DOI:** 10.1002/ece3.7810

**Published:** 2021-06-27

**Authors:** David A. Roshier, Johannes Signer, Andrew Carter

**Affiliations:** ^1^ Australian Wildlife Conservancy Subiaco East WA Australia; ^2^ School of Animal and Veterinary Science University of Adelaide Adelaide SA Australia; ^3^ Wildlife Sciences Faculty of Forest Science and Forest Ecology University of Goettingen Göttingen Germany; ^4^ Institute for Land, Water and Society Charles Sturt University Albury NSW Australia

**Keywords:** animal movement, fox management, GPS tracking, habitat selection analysis, mesopredators, recursion

## Abstract

The introduced red fox (*Vulpes vulpes*) now occupies most of the Australian continent outside the tropics, including arid and semiarid ecosystems. Information on the water requirements of foxes is scant, but free water is not thought to be required if adequate moisture‐containing food is available. The frequency and duration of visits by foxes fitted with GPS collars to known artificial watering points in semiarid Australia were recorded for 22 individual foxes across four austral seasons between October 2015 and November 2017, providing >93,000 location fixes. We modeled home range and the distance traveled by range‐resident foxes beyond their home range to reach known water sources. We used recurse analysis to determine the frequency of visitation and step‐selection functions to model the speed and directionality of movement inside and outside the home range. Our study demonstrates that some foxes in this semiarid environment utilize free‐standing water. The findings suggest that artificial watering points can be used as a focal point for conducting strategic fox control in arid and semiarid environments. Additionally, strategies that restrict access to water by foxes may reduce their duration of occupancy and/or long‐term abundance in parts of the landscape, thus providing benefits for conservation and agriculture.

## Graphical abstract

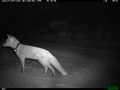

## 1 INTRODUCTION

Understanding the need to drink and the role of surface water in sustaining canids is potentially a key aspect in the conservation and management of this diverse and widespread group of predators. Human–predator conflicts and competitive interactions between sympatric canids can be exacerbated by the addition of artificial water sources (Arjo et al., 2007; Kluever & Gese, 2016, 2017; Kluever et al., 2017). The presence of artificial water sources can also have secondary impacts on prey species through the presence of predators (e.g., Harrington et al., 1999). European settlement of Australia's arid and semiarid zone in the late 1800s brought with it widespread establishment of artificial watering points for livestock (James et al., 1999). This development has wrought profound changes to the region's flora and fauna (reviewed by James et al., 1999) and may facilitate the presence of introduced predators such as the European red fox (*Vulpes vulpes*).

Red foxes were introduced successfully to Australia in the early 1870s (Rolls, 1984) and now inhabit the entire Australian mainland south of the tropics (Saunders et al., 1995). In the period since, Australia has experienced the highest recent mammal extinction rate in the world, with most “critical weight range” species (i.e., 35–5,500 g; Burbidge & McKenzie, 1989) now extinct, either regionally or globally (Moseby, 2012; Woinarski et al., 2014). Predation by foxes is regarded as a major driver of the decline and extinction for many of these species (Dickman, 1996; Fleming et al., 2014; Woinarski et al., 2015, 2019), as well as having impacts on the composition and diversity of communities of small terrestrial vertebrates (mammals and reptiles) (Moseby et al., 2009; Roshier et al., 2020).

In temperate and continental climes in the northern hemisphere, it is considered unlikely that free water is necessary for survival in foxes if preformed water is available in the diet (Haltenorth & Roth, 1968; Sargeant, 1978; Uraguchi & Takahashi, 1998). In Australia, it has also been assumed that free water is not normally required by foxes (e.g., Wallach et al., 2009), although the presence of dingoes around artificial water points has been shown to modify the presence of introduced predators (Brawata & Neeman, 2011). This suggests some dependence on free water and there is evidence that foxes drink in this environment—at least occasionally. In a study in arid New South Wales (NSW), a single fox fitted with a tracking collar traveled to an artificial water source to drink (Marlow, 1992). Additionally, a study on field metabolic rates and water turnover in foxes in temperate NSW concluded that during hot weather foxes likely supplemented their dietary water intake by drinking (Winstanley et al., 2003).

Here, we document visitation of artificial watering points in semiarid NSW, Australia, by foxes fitted with GPS collars spanning four austral seasons between October 2015 and November 2017. This environment is markedly different to most habitats and climates of its natural range in North America and Europe and we would expect the biophysical conditions to be constraining, at least some of the time. In addition, we model the rate and directionality of movement using step‐selection functions inside the home range with extraterritorial excursions to water. Our results have implications for the management of artificial watering points in arid and semiarid landscapes, for improving fox management, and for managing fox impacts.

## 2 MATERIALS AND METHODS

### 2.1 Study area

**FIGURE 1:**
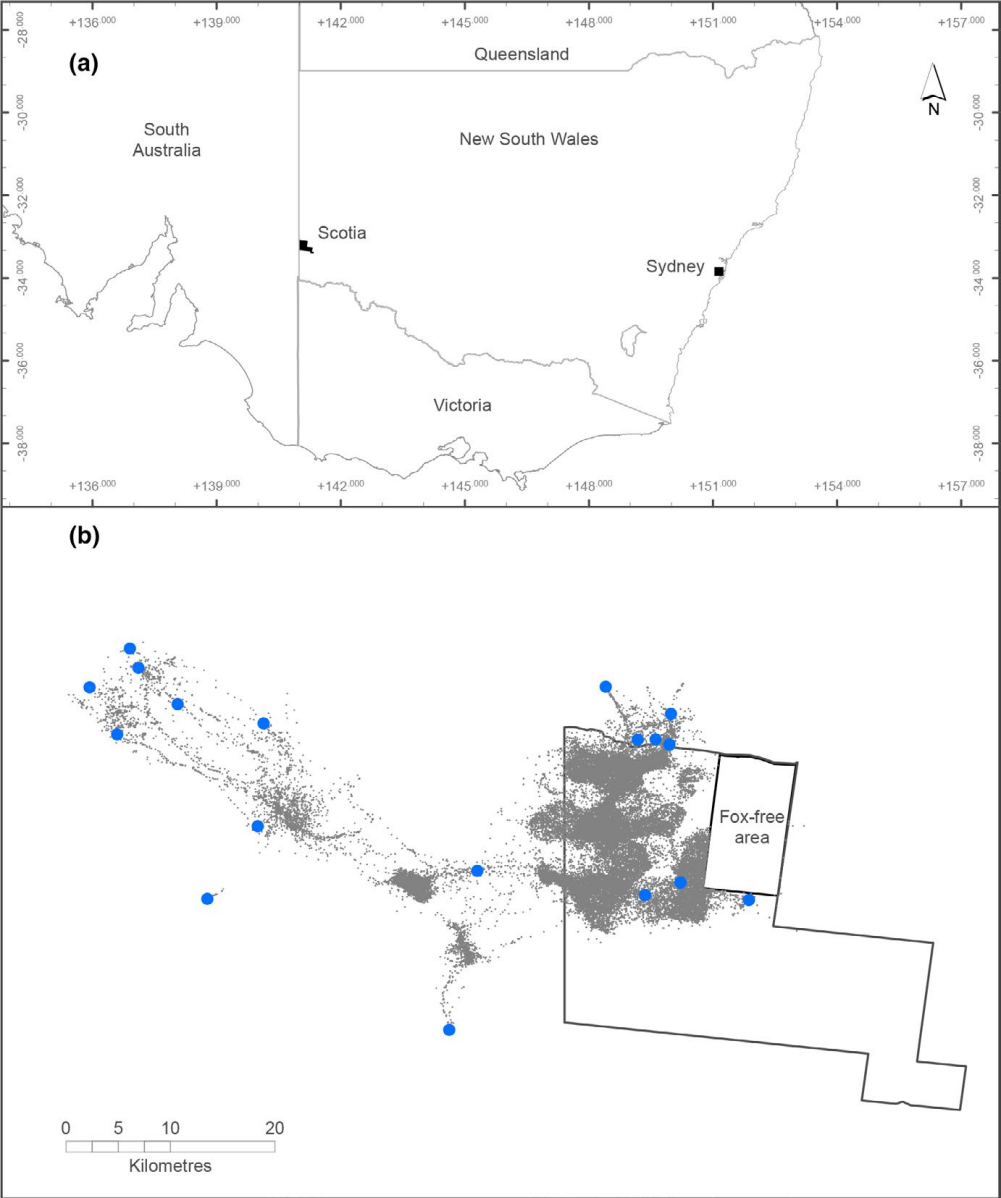
Location of Scotia Wildlife Sanctuary within Australia (a), plus known artificial watering points on and around Scotia (blue circles) and GPS location fixes (gray dots, *n* = 93,969) for 22 foxes tracked from 2015 to 2017 (b)

Our study was conducted at Scotia Wildlife Sanctuary, a 64,659‐ha private conservation reserve in south‐western New South Wales, Australia (−33.15°S, 141.06°E; Figure 1) owned and managed by Australian Wildlife Conservancy. The climate is semiarid with low and highly variable rainfall (spatially and temporally) that averages 250 mm per year with high evapotranspiration (1,500 mm per year) and low relative humidity (average: 20%) (Australian Wildlife Conservancy, unpublished data). The site is characterized by cool winters (average maximum temperature: 17°C) and hot summers (average maximum temperature: 30℃), with annual temperature extremes ranging from –6℃ to 48℃. Few drainage features are present across the mostly flat broader landscape, which is dominated by extensive dune fields. Vegetation is predominantly *Eucalyptus* multi‐stemmed (“mallee”) open‐shrubland with a *Triodia scariosa* (“spinifex”) or shrubby understorey, on the dunes, and *Casuarina pauper* woodland on the swales (Westbrooke et al., 1998).

Scotia Wildlife Sanctuary incorporates an 8,000 ha fenced area from which all introduced predators have been removed and to which a suite of regionally extinct mammals have been reintroduced (Kanowski et al., 2018). The present study was conducted outside the fenced area. At Scotia, the mallee vegetation with spinifex understorey has a higher abundance and diversity of small terrestrial vertebrates (predominantly reptiles), while most of the small to medium mammals are regionally extinct apart from one Murid and four Dasyurid species (Roshier et al., 2020). All small native mammals occur at low abundance (unpublished data). Other prey species present that have been recorded in fox diets previously include the introduced house mouse *Mus musculus*, European rabbit *Oryctolagus cuniculus,* feral goat *Capra hircus*, ground‐nesting birds, and insects (Lugton, 1993; White et al., 2006).

Foxes were trapped (details below) across an area of approximately 19,000 ha where no lethal control had occurred for more than 6 years prior to this study. No permanent free water is available within the study area, although during our study two decommissioned artificial dams (ponds) held water periodically. Additional artificial watering points are available on neighboring properties adjoining the study area (Figure 1), and water is also available sporadically from puddles formed on roads and depressions following heavy rainfall, although these rarely last for more than a day or two. As part of related research (Carter et al., 2019), 72 camera traps (HC600, Reconyx) were placed throughout the study area, operating for the first 25 days of each month for the duration of this study. Records of the frequency and duration of puddle formation following rainfall events were recorded by inspecting the images captured at a site known to pond surface water in shallow puddles.

### 2.2 Fox capture and handling

Foxes were trapped in three consecutive years during the following months: October–November (2015); July–August (2016); and June–July (2017). Foxes were caught using two types of traps: (a) custom‐made box traps that were buried in the ground with an entrance resembling the burrow of a rabbit and a trap door activated by a treadle plate, or (b) #1.5 Victor Soft‐Catch™ foot‐hold traps (Woodstream Corporation) fitted with in‐line springs and swivels, tethered with chain to two steel stakes (500 mm long) driven below ground‐level beneath the trap. A variety of attractants were used including chicken pieces, rabbit meat, fox scats, and commercial scent lures. Traps were checked for captures at dawn and dusk, with trapped foxes subdued using either an animal‐restraining pole (Ketch‐all™) and blankets (when in foot‐hold traps), or by being transferred from box traps into a wire crush cage (Wiretainers Pty Ltd), before being anesthetized with an intramuscular injection of a Tiletamine/Zolazepam combination (Zoletil 100^®^; Virbac Pty Ltd) at a rate of 8–10 mg/kg body mass. All methods conformed to Standard Operating Procedures for fox trapping in Australia (Sharp, 2016a, 2016b).

Sedated foxes were weighed, sexed, inspected for injuries and condition, and then fitted with a GPS collar (Quantum 4000E; Telemetry Solutions) that weighed 170 g (±5 g, *SD*), which was <5% of the foxes’ bodyweight. GPS units were programmed to operate for the first 25 days of each month, recording location fixes at 20‐min intervals between 17:00 hr and 09:00 hr and at 96‐min intervals between 09:00 hr and 17:00 hr. Units were programmed to search for satellites for a maximum of 60 s at each time interval. Collars contained remote drop‐off mechanisms that were activated after approximately 4 months of data collection. The only injuries sustained by foxes during trapping were localized swelling and minor abrasions of the foot compressed in the foot‐hold trap. No injuries were considered likely to affect foxes post‐release, and after receiving their collars, foxes were placed in the nearest available cover and monitored to ensure recovery from anesthesia.

### 2.3 Data filtering

We used ad hoc opportunities to determine the accuracy of GPS collars in the study area. After drop‐off mechanisms were activated, collars remained in the study area at a fixed location until recovered. For each of 10 collars (2 collars in year 1; 5 collars in year 2; 3 collars in year 3), accuracy was assessed from 106 location attempts (i.e., the equivalent of two full days of data collection) using the same collection schedule as collars placed on active foxes (see Roshier & Carter, 2021 for additional detail). All fixes in the primary dataset (*n* = 95,413) were managed in Movebank (Kranstauber et al., 2011). The data were filtered by removing locations with HDOP = 9.9 (max. value) and elevations >200 meters above mean sea level and negative values of mean sea level, noting that the terrain is generally flat and elevations range from approximately 30–100 m across the study area. We then removed location fixes for which the rate of movement from the previous fix was >5 m/s and/or the turning angle was between 166° and 194° (see Roshier & Carter, 2021 for additional detail).

### 2.4 Artificial watering point visitation

Artificial watering points situated beyond the study area were identified by gathering information from neighboring land managers or reviewing topographic maps and aerial photography. We used the *recurse* package (v. 1.1.0, Bracis, 2018) in R (v. 3.5.2, R Core Team, 2020) to determine the date, time, and frequency of visits to watering points. The *recurse* package enables users to specify a circle of a particular radius at locations of interest. It then counts the number of trajectory segments of the movement paths of one or many individuals that intersect the circle. Each such intersection is classified as one visit. The package uses linear interpolation to estimate the entrance and exit times and calculates other metrics such as visit duration and time since previous visit. For this analysis, we used a radius of 500 m. Excursions to water to drink are often brief and the radius selected needed to be sufficiently large to detect an animal in the 20 min between GPS location fixes. Given the highly variable rainfall in the study area, it was not possible to be certain of the status of each watering point over the three years of the study and whether it contained water at the time of visit by a fox. We have interpreted the data accordingly.

To determine whether such excursions to water points occurred at night or in daylight, we used the *crespuscule* function in the *maptools* package (ver. 1.0‐2, Lewin‐Koh et al., 2011) in R (v. 4.0.3, R Core Team, 2020). We used the period from first light to last light to classify as daylight. We used civil twilight and a solar depression angle of 6° to determine first light and last light at each location. All times are Australian Eastern Standard Time and take no account of daylight‐saving time.

### 2.5 Home range estimation and modeling of movement

For this analysis, we wanted to determine how far, and often, individual foxes ventured beyond their current home range to visit artificial watering points. We used the continuous time movement modeling (c*tmm*) package (ver. 0.5.9, Fleming et al., 2019) in R (v. 3.6.2, R Core Team, 2020) to model an animal's home range. We determined whether individuals were range resident or nonsedentary by fitting a semivariance function to the data (see Roshier & Carter, 2021 for additional detail). Only data from those individuals that were range resident were used to model rates of movement and directionality using step‐selection functions.

### 2.6 Habitat selection and movement

We used integrated step‐selection functions (iSSA; Avgar et al., 2016; Fieberg et al., 2021) to model habitat selection and movement of foxes. The iSSA method compares covariates at the end point of each realized step (a straight line connecting consecutive relocations of individual foxes) with a set of control steps. The control steps were generated by fitting distributions to the observed step lengths and relative turning angles. We fitted a gamma distribution and a von Mises distribution to the step lengths and turning angles, respectively. We pooled steps of all animals for fitting tentative distributions, but allowed individual‐specific deviations in the model. Including attributes of the steps (cosine of the turn angle and natural logarithm of the step length) and interactions between these attributes and covariates, allows to correct tentative parameter estimates of the distributions and to model speed and directionality for different covariate values. We were interested whether foxes select for water sources relative to their home range. Hence, we included two covariates: the natural log of the distance to the next water source and whether or not a given location was within or outside the home range of each individual fox. In order to model differences in movement directionality and speed inside and outside of an individuals’ home range, we further included interactions between the cosine of the turning angle or the step length, and whether or not a step started inside or outside of the home range (Signer et al., 2019). To account for individual variation, we used mixed‐effects models for step selection functions following Muff et al., 2020. We used random slopes for covariates of habitat selection (log distance to the next water source and home range, and interaction thereof) and covariates for movement behaviors (log of step length, cosine of turn angle, and interaction of whether a pixel was within the home range or not). Home range is used as an indicator variable with values 0 (pixels outside the home range) and 1 for pixels inside the home range. For these analyses, we used R packages *raster* (ver 3.4–5; Hijmans, 2020), *amt* (ver 0.1.4; Signer et al., 2019), and *glmmTMB* (ver 1.0.2.1; Brooks et al., 2017) with Program R (ver 4.0.3; R Core Team, 2020).

## 3 RESULTS

### 3.1 Weather

Annual rainfall at Scotia from 2015 to 2017 was near average, at 235, 273, and 237 mm, respectively. Monthly rainfall and average temperatures for the period when foxes were tracked are provided in Table 1. Camera‐trap monitoring at a site known to form puddles after heavy rainfall identified six occasions when surface water puddles were available while foxes were tracked (November 2015; January, August, September, November 2016; October 2017). On each occasion, these puddles dried quickly, persisting for <12 hr after first being observed.

**TABLE 1:** Monthly rainfall plus average daily maximum and minimum temperatures (interpolated data) for Scotia Wildlife Sanctuary during the period when foxes were tracked with GPS collars

	Winter	Spring			Summer			Autumn
	Aug	Sep	Oct	Nov	Dec	Jan	Feb	Mar
Rainfall (mm)								
2015	–	–	9	8	0	–	–	–
2016	23	89	9	11	11	29	1	16
2017	23	2	12	63	–	–	–	–
Ave. max. temp (°C)^a^								
2015	–	–	32	31	35	–	–	–
2016	19	19	24	29	32	34	34	32
2017	23	24	27	30	–	–	–	–
Ave. min. temp (°C)^a^								
2015	–	–	13	15	18	–	–	–
2016	6	8	10	12	17	19	17	17
2017	6	8	12	15	–	–	–	–

^a^ Data from Raupach et al. (2009, 2012).

### 3.2 Fox GPS telemetry

We tracked 22 foxes (9 ♂, 13 ♀) spanning four austral seasons between October 2015 and November 2017 (Table 2). Four foxes (1 ♂, 3 ♀) were tracked in two consecutive years, and hence, we collected 26 tracking datasets in total.

**TABLE 2:** Summary of tracking data for foxes at Scotia Wildlife Sanctuary (2015–2017), including whether the individual was range resident (yes/no) and the area of home range (after Roshier & Carter, 2021) and records of visitation to putative, artificial watering points

Fox ID	Sex	Weight (kg)	First tracked	Last tracked	Tracking period (days)	No. GPS locations	Range resident	AKDE95 home range area (ha)	No. visits to watering points^a^
AM663	♂	4.8	8/10/2015	2/02/2016	117	4,395	Yes	2033	22 (1)
AM546	♂	4.0	14/10/2015	25/01/2016	103	4,003	No	NA	5 (1)
AF266	♀	4.0	1/12/2015	11/03/2016	101	4,032	Yes	1,455	17 (2)
AF546	♀	5.0	9/11/2015	4/12/2015	25	974	No	NA	0
AF549	♀	4.0	20/10/2015	11/02/2016	114	4,267	Yes	1,680	18 (2)
AF636	♀	3.4	24/11/2015	25/01/2016	62	2,204	No	NA	11 (2)
AF963‐15	♀	4.0	24/11/2015	11/03/2016	108	3,792	Yes	1,037	0
AF232‐A	♀	3.6	1/08/2016	14/11/2016	105	4,519	No	NA	11 (2)
AF232‐B	♀	3.6	1/08/2016	24/11/2016	115	5,129	No	NA	37 (4)
AM262	♂	4.8	1/08/2016	3/09/2016	33	1,419	No	NA	0
AF228	♀	4.7	1/08/2016	22/08/2016	21	837	No	NA	0
AF369‐16	♀	4.6	1/08/2016	24/11/2016	115	3,719	Yes	1,466	1 (1)
AF569‐16	♀	3.8	1/09/2016	22/12/2016	112	4,043	Yes	1729	64 (1)
AM654‐16	♂	4.4	1/08/2016	24/11/2016	115	5,150	Yes	1,341	10 (2)
AM734	♂	4.4	1/08/2016	25/11/2016	116	5,109	No	NA	10 (2)
AF825	♀	3.6	1/09/2016	24/12/2016	114	4,207	Yes	1733	0
AF963‐16	♀	3.7	1/08/2016	22/08/2016	21	1,148	No	NA	0
AM366	♂	5.4	1/08/2017	21/11/2017	112	5,052	Yes	2,282	1 (1)
AM376	♂	4.6	1/08/2017	27/10/2017	87	3,928	Yes	1,211	0
AF369‐17	♀	4.4	1/08/2017	15/08/2017	14	740	No	NA	0
AF536	♀	4.1	1/08/2017	31/10/2017	91	3,927	Yes	735	0
AF569‐17	♀	3.6	1/08/2017	27/10/2017	87	3,158	No	NA	56 (3)
AM536	♂	4.3	1/08/2017	20/11/2017	111	4,704	No	NA	21 (2)
AM568	♂	3.4	1/08/2017	20/11/2017	111	5,292	No	NA	98 (7)
AM654‐17	♂	4.9	1/08/2017	31/10/2017	91	4,235	Yes	2,537	25 (3)
AF767	♀	4.0	1/08/2017	27/10/2017	87	3,986	Yes	2,196	83 (3)

^a^ Figures in parenthesis are the number of watering points visited.

### 3.3 Artificial watering point visitation

**FIGURE 2:**
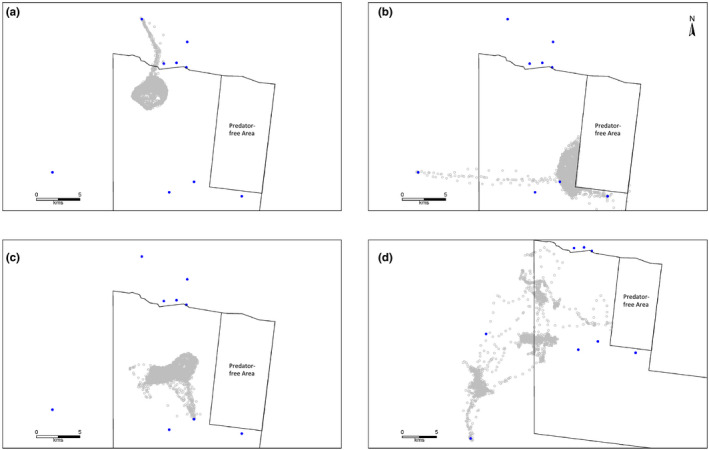
Data from GPS collars (gray circles) fitted to four individual foxes that show movements to visit watering points (blue dots). (a) and (b) is females (IDs AF266 (range resident) and AF569‐17, respectively), while (c) and (d) is male (IDs AM663 (range resident) and AM546 (nonsedentary), respectively)

Visits to artificial watering points were identified for 15 individual foxes, both range resident and nonsedentary, noting that four individuals were tracked in two consecutive years (Table 2). Of the 13 range‐resident foxes, seven individuals only ever visited sites outside their (AKDE95) home range, two individuals visited sites both inside and outside their home range, and four individuals had no instance of visiting an identified water source (Table 3). Extraterritorial trips to watering points resulted in range‐resident foxes traveling a minimum (Euclidean) distance of between 1.0 and 5.7 km each way (mean ± *SD*, 3.0 ± 1.7 km) beyond their home‐range boundary to reach those sites, including two individuals that traveled >5 km each way (Figure 2). Another female fox (AF569‐17), whose home‐range boundary could not be modeled adequately using AKDE, on two occasions made directed trips of 14.6 km each way during spring to access a water source on a neighboring property (Figure 2b).

**TABLE 3:** The number of visits to artificial watering points by *range‐resident* foxes at Scotia Wildlife Sanctuary (2015–2017) by month, including the average interval in days between visits (range is shown in parenthesis). These data were derived from the *recurse* package and intervals of <1 represent revisits where the animal has left the immediate vicinity (>500 m) of the water source and returned sometime during the same day. Empty cells indicate no GPS tracking data were available. For each month, use of watering points *outside* the home range (AKDE95) area are identified by *orange* cells; use of watering points *inside* the home range only are signified by *blue* cells; and *green* cells signify use of watering points both *outside and inside* the home range

		Aug		Sep		Oct		Nov		Dec		Jan		Feb		Mar	
	Fox ID	No. visits	Interval	No. visits	Interval	No. visits	Interval	No. visits	Interval	No. visits	Interval	No. visits	Interval	No. visits	Interval	No. visits	Interval
2015 Captures	AM663					2	4	5	3.2 (<1–5.9)	11	2.2 (1.2–3.9)	4	5.0 (3.1–6.9)	0^a^			
	AF266									6	4.2 (<1–12.0)	5	1.5 (<1–2.0)	5	4.2 (2.9–6.1)	1	
	AF549					1		2	1.9	7	2.8 (1.0–7.0)	7	3.0 (1.0–8.1)	1			
	AF963‐15							0^a^		0		0		0		0	
2016 Captures																	
	AF369‐16	0		0		0		1									
	AF569‐16			16	1.2 (<1–3.9)	8	3.1 (1.9–5.1)	20	1.1 (<1–2.3)	20	1.0 (<1–1.9)						
	AM654‐16	9	1.5 (<1–3.3)	1		0		0									
	AF825			0		0		0		0							
2017 Captures	AM366	0		0		0		1									
	AM376	0		0		0											
	AF536	0		0		0											
	AM654‐17	4	0.2 (<1–0.5)	7	1.8 (<1–6.9)	14	2.1 (<1–13.6)										
	AF767	21	1.1 (<1–2.9)	31	0.8 (<1–4.0)	31	0.8 (<1–4.8)										

^a^ GPS data available for 2 days only.

Excluding foxes that were never recorded at watering points, on average, each individual visited two different watering points (range, 1–7) on 29 separate occasions (range, 1–98) (Table 2). The number of records at watering points by individual foxes during a calendar month varied from 0 to 34 visits, with intervals between those visits ranging from <1 day to 12 days (Table 3). For range‐resident foxes, no patterns were evident with regard to frequency of visitation, across study months, in any of the three years. Likewise, there was little consistency in whether foxes were using extraterritorial watering points or not. In 2015, all visitations to water sources were extraterritorial, while in 2016 and 2017 some foxes used only extraterritorial watering points, while others used water sources both inside and outside their territory or a combination of both, depending on month (Table 3).

### 3.4 Timing and duration of visits to artificial watering points

**FIGURE 3:**
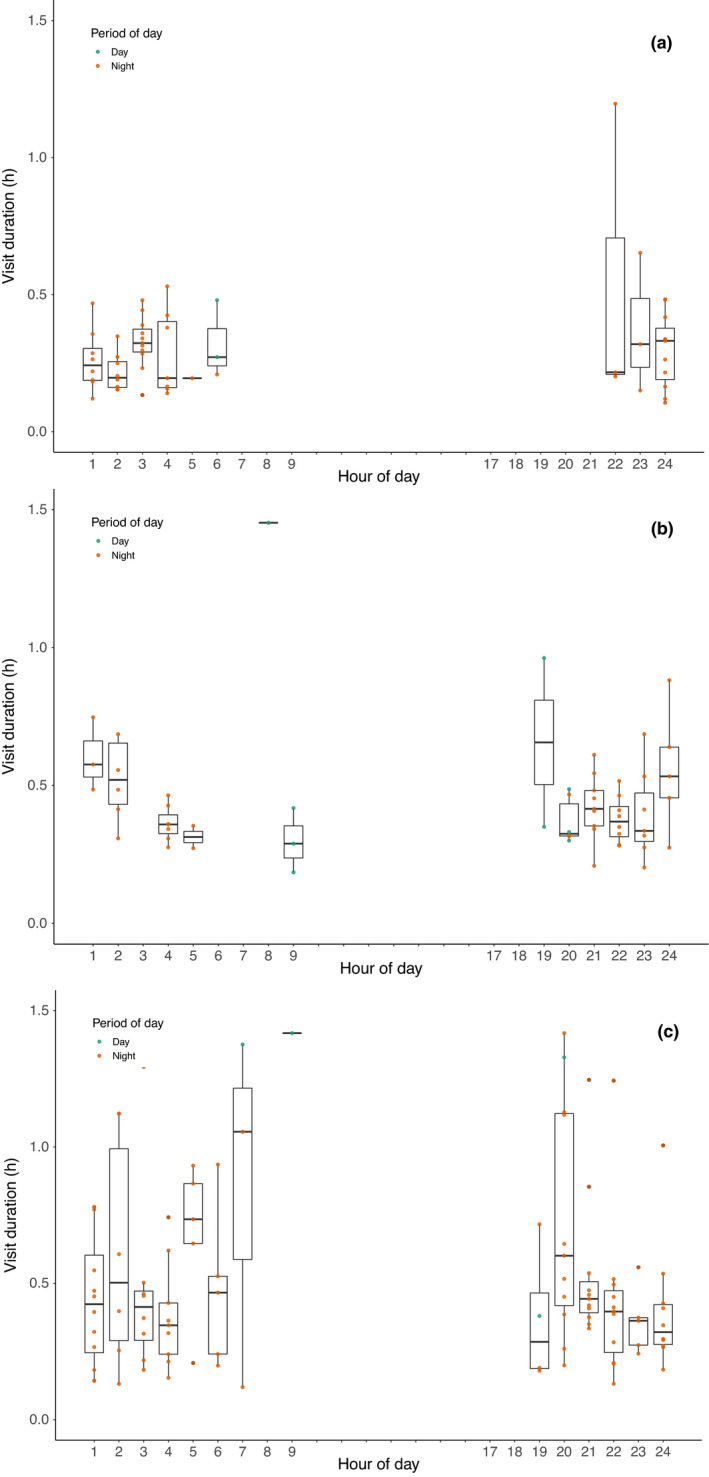
Duration of visitation to artificial watering points, according to hour of the day, by range‐resident foxes fitted with GPS collars at Scotia Wildlife Sanctuary: (a) foxes collared in 2015, (b) 2016, (c) 2017. Note: ticks on *x*‐axis with no label signify no data

In each year, most visitations of watering points were made at night. Foxes captured in 2015 visited watering points 55 times (2 day, 53 night; noting that “night” is last light to first light) spending an average of 0.3 hr (range, 0.1–1.2) at the site during each visit. Foxes visited watering points in all hours between 22:00 and 06:00 (Figure 3a). In 2016, foxes were recorded 59 times at watering points (9 day, 50 night), spending an average of 0.4 hr (range = 0.1–1.45) at the site during each visit. In this second year of the study, location fixes at watering points were recorded in the hours between 19:00 and 09:00 (Figure 3b)—a broader range of times compared to the previous year. In 2017, there were 109 records at watering points (4 day, 105 night), with foxes spending an average of 0.7 hr (range, 0.1–4.9) at the site during each separate occasion. Similar to the previous year, foxes visited watering points in all hours between 19:00 and 08:00 (Figure 3c). There was no strong evidence that in any of the three years, duration of visits was influenced by time of the day.

### 3.5 Habitat selection and rates of movement

**FIGURE 4:**
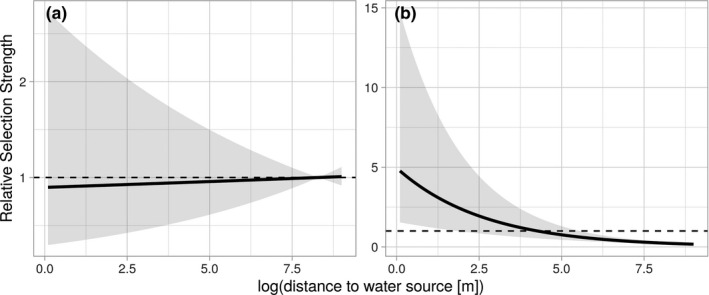
Population level relative selection strength for water sources of range‐resident foxes, inside (a) and outside (b) their home range (AKDE95). Black line shows population level strength of selection, and gray area show 95% confidence intervals. The mean distance to a non‐permanent, artificial water source is ~4 km for a fox located inside its home range

**FIGURE 5:**
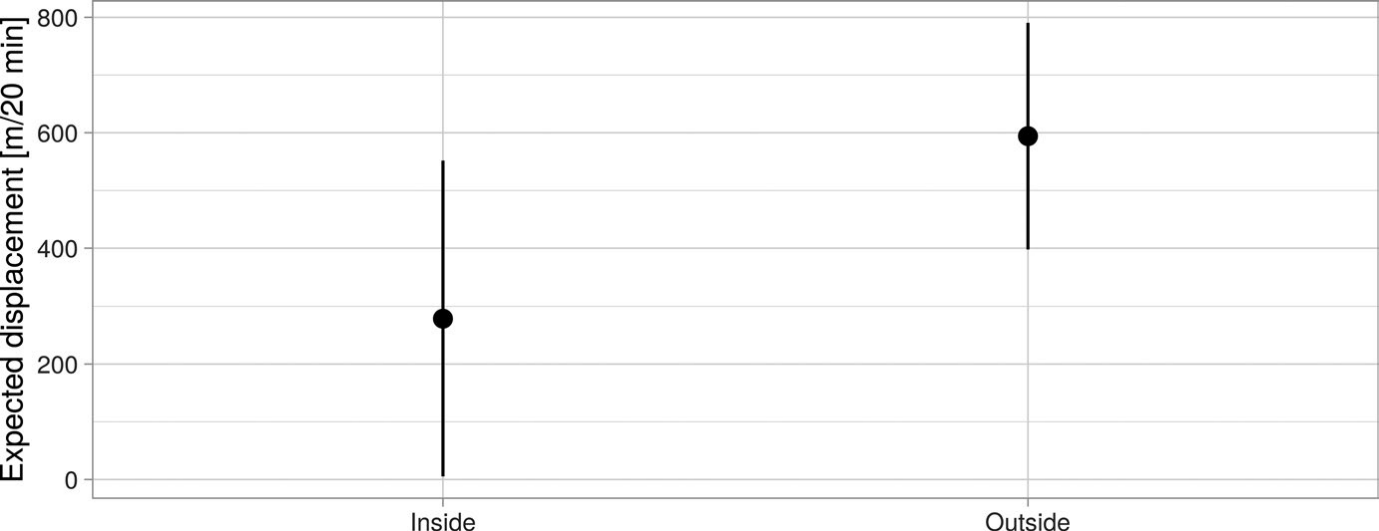
Mean rate of movement (estimate (m/20 min) ±95%CL) for foxes inside and outside their home range (AKDE95)

In this analysis the only resource or habitat in the models was artificial, non‐permanent water sources and whether the current location was inside or outside the home range (AKDE95). Both habitat selection and movement were significantly influenced by distance to water sources and whether or not a fox was within its home range (Table 4). At the population level, foxes showed no selection for pixels closer to water when inside their home range (Figure 4a), but strong selection for pixels closer to water sources when outside their home range (Figure 4b). Movements inside the home ranges were less directed (the coefficient for the cosine of the turn angle inside the home is negative, this means that the concentration parameter of the turn‐angle distribution is smaller and thus movement is less directed; Table 4). In addition, movement rates approximately doubled outside home ranges from an average displacement of 279 m/20 min inside the home range to 594 m/20 min outside of the home range (the interaction between log(step length) and the home range indicator was significant, see Table 4 and Figure 5).

**TABLE 4:** Estimated coefficients of the integrated step‐selection function inside and outside the home range (AKDE95). Effects with a *p*‐value of <0.05 were considered as significant

Variable	Estimate	Std. Error	*p*‐value
home range (hr)	−1.70864	0.53169	0.001
log(distance to water)	−0.37485	0.06790	<0.001
log(distance to water):hr	0.38799	0.06186	<0.001
cos(turn angle)	0.30357	0.09997	0.002
cos(turn angle):hr	−0.28146	0.10084	0.005
log(step length)	0.45619	0.14541	0.002
log(step length):hr	−0.45863	0.14492	0.002

## 4 DISCUSSION

Our 3‐year study, that tracked 22 foxes in semiarid Australia, showed that some individuals utilize free‐standing water—sometimes traveling many kilometers beyond their established territories to visit watering points. This confirms that, like other members of the Canid family (Bothma, 1998), red foxes may supplement their dietary water intake by drinking free water when it is available. Given the distance traveled each way and the frequency with which some individuals used extraterritorial water sources, at certain times the requirement for free water appears acute. For example, in the summer of 2015/16, one adult female (AF266) made return trips in excess of 11 km to an earth dam (storage) holding water, every 3 days on average (Table 3). While we cannot confirm that all water points contained water at the time of visitation, the directed nature of extraterritorial trips (Figure 2), the short duration of visitation (Figure 3), the directed mode of travel (Table 4), and the faster rate of travel once outside their home range or territory (Figure 5) suggest they did contain water. Moreover, there is no other probable reason for foxes to leave their territory to visit these sites in this manner, other than to drink.

Nine foxes were never recorded at artificial watering points, although the monitoring period for five of these individuals was <35 days—substantially shorter than for all other foxes (Table 2). It is possible that some collared foxes visited watering points during the day when location fixes were collected at lower frequency or that some individuals did not travel to water because they had access to other water sources unknown to us, although both possibilities seem improbable. A more likely explanation is that these foxes supplemented their dietary water intake, whenever possible, by utilizing surface water puddles which were known to be present for short periods throughout the study after heavy rainfall. Alternatively, it is possible that the foxes in our study that were never recorded at artificial watering points consumed dietary items with a higher water content than those foxes that did travel to water. For example, Dell'Arte and Leonardi (2009) concluded that foxes in arid Tunisia selectively fed on prey rich in water, primarily beetles, to compensate for the shortage of free water in that environment. However, such a strategy requires a increase in prey consumption. In a study of two desert‐dwelling canids in North America, the coyote *Canis lantrans* and kit fox *Vulpes macrotis*, the acquisition of sufficient water required a substantial increase in prey consumption compared to that required to meet energy needs and only the smaller kit fox could survive without free water (Golightly & Ohmart, 1984). In contrast, in arid Australia the dingo *Canis dingo* requires access to water every few days (Allen, 2012). This suggests that there is an interplay between physical size, prey availability, water availability and climate that determines whether canids need to drink and, therefore, require access to free water. Thus, the need to drink may be species specific, condition dependent, and local.

In many cases, extraterritorial trips to artificial watering points followed almost identical directed movement paths (Figure 2) which indicates that foxes use spatial memory to revisit watering points, as has been suggested for other vertebrates (e.g., Polansky et al., 2015). Furthermore, our results imply that foxes have a detailed understanding of landscape features that are situated well beyond their territory (i.e., a high perceptual range: Lima & Zollner, 1996). The source of this understanding in foxes is unclear, but possibilities include prior experiences at particular locations (e.g., Bracis & Mueller, 2017; Van Moorter et al., 2009), communication with conspecifics (e.g., Peters & Wozencraft, 1989), or by olfactory means (e.g., Jacobs, 2012).

Our results demonstrate that in this semiarid environment artificial watering points provide resources for a large proportion of the fox population, which underscores the value of considering water use by canids when developing management strategies in this landscape (Brawata & Neeman, 2011). Specifically, poison baiting with sodium fluoroacetate is currently the most widely practiced form of broad‐scale fox control in Australia (Saunders et al., 2010) and bait placement is known to affect uptake rates by foxes considerably (Carter & Luck, 2013; Trewhella et al., 1991). Conducting targeted fox baiting within the vicinity of watering points in arid and semiarid areas may therefore improve bait uptake and increase the efficacy of fox‐baiting programs for protecting wildlife and livestock. Particularly if such control activities were timed to coincide with periods of rainfall deficit or extended periods of heat accumulation. Likewise, the effectiveness of other forms of fox control, such as trapping and shooting, may also increase if targeted around watering sites in these environments. Moreover, artificial watering points have been shown to support higher activity and density of prey species (James et al., 1999; Valeix et al., 2010), meaning there may be a higher risk of predation by foxes at these sites (Davies et al., 2010). Hence, strategies that restrict access to water by foxes may reduce their duration of occupancy and/or long‐term abundance in parts of the landscape and lessen their impact on native prey species and livestock by decreasing predation around artificial watering points.

## CONFLICTS OF INTEREST

The authors declare no conflicts of interest.

## AUTHOR CONTRIBUTION

**David Roshier:** Conceptualization (equal); Formal analysis (equal); Writing‐original draft (equal); Writing‐review & editing (equal). **Johannes Signer:** Formal analysis (equal); Writing‐original draft (supporting); Writing‐review & editing (supporting). **Andrew Carter:** Conceptualization (equal); Formal analysis (equal); Writing‐original draft (equal); Writing‐review & editing (equal).

## Data Availability

The data are archived at the Movebank Data Repository (https://www.datarepository.movebank.org) as “Carter and Roshier (2019).” https://doi.org/10.5441/001/1.72hh609t.
